# Farming for Ecosystem Services: An Ecological Approach to Production Agriculture

**DOI:** 10.1093/biosci/biu037

**Published:** 2014-04-08

**Authors:** G. Philip Robertson, Katherine L. Gross, Stephen K. Hamilton, Douglas A. Landis, Thomas M. Schmidt, Sieglinde S. Snapp, Scott M. Swinton

**Affiliations:** G. Philip Robertson (robert30@msu.edu) is a professor at the Kellogg Biological Station (KBS) and in the Department of Plant, Soil, and Microbial Sciences at Michigan State University (MSU), in East Lansing. Katherine L. Gross is a professor at KBS and in the Department of Plant Biology at MSU. Stephen K. Hamilton is a professor at KBS and in the Department of Zoology at MSU. Douglas A. Landis is a professor in the Department of Entomology at MSU. Thomas M. Schmidt is a professor in the Department of Ecology and Evolutionary Biology at the University of Michigan, in Ann Arbor. Sieglinde S. Snapp is a professor at KBS and in the Department of Plant, Soil, and Microbial Sciences at MSU. Scott M. Swinton is a professor in the Department of Agricultural, Food, and Resource Economics at MSU. All of the authors are lead investigators with the KBS Long-Term Ecological Research Program.

**Keywords:** agriculture, ecosystem services, biocontrol, water quality, greenhouse gas mitigation

## Abstract

A balanced assessment of ecosystem services provided by agriculture requires a systems-level socioecological understanding of related management practices at local to landscape scales. The results from 25 years of observation and experimentation at the Kellogg Biological Station long-term ecological research site reveal services that could be provided by intensive row-crop ecosystems. In addition to high yields, farms could be readily managed to contribute clean water, biocontrol and other biodiversity benefits, climate stabilization, and long-term soil fertility, thereby helping meet society's need for agriculture that is economically and environmentally sustainable. Midwest farmers—especially those with large farms—appear willing to adopt practices that deliver these services in exchange for payments scaled to management complexity and farmstead benefit. Surveyed citizens appear willing to pay farmers for the delivery of specific services, such as cleaner lakes. A new farming for services paradigm in US agriculture seems feasible and could be environmentally significant.

**Row-crop agriculture is one of the most extensive** and closely coupled natural–human systems and has extraordinary implications for human welfare and environmental well-being. The continued intensification of row-crop agriculture provides food for billions and, for at least the past 50 years, has slowed (but not stopped) the expansion of cropping onto lands valued for conservation and other environmental services. Nevertheless, intensification has also caused direct harm to the environment: The escape of reactive nitrogen and phosphorus from intensively managed fields pollutes surface and coastal waters and contaminates groundwater, pesticides kill nontarget organisms important to ecological communities and ecosystems sometimes far away, soil loss threatens waterways and long-term cropland fertility, accelerated carbon and nitrogen cycling contribute to climate destabilization, and irrigation depletes limited water resources.

The search for practices that attenuate, avoid, or even reverse these harms has produced a rich scientific literature and sporadic efforts to legislate solutions. That these harms persist and, indeed, are growing in the face of increased global demands for food and fuel underscores the challenge of identifying solutions that work in ways that are attractive to farmers and responsive to global markets. On one hand are farmers’ needs for practices that ensure a sustained income in the face of market and consumer pressures to produce more for less; on the other are societal demands for a clean and healthful environment. Most growers are caught in the middle.

One avenue for addressing this conundrum is the potential for row-crop producers to farm for more than food, fuel, and fiber. Growing recognition that agriculture can provide ecosystem services other than yield (Swinton et al. 2007, Power 2010) opens a potential for society to pay for improvements in services provided by farming: a clean and well-regulated water supply, biodiversity, natural habitats for conservation and recreation, climate stabilization, and aesthetic and cultural amenities such as vibrant farmscapes.

Operationalizing such an enterprise, however, is far from straightforward: Farming for services requires knowledge of what services can be practically provided at what cost and how nonprovisioning services might be valued in the absence of markets. The costs of providing services are both direct (e.g., the cost of installing a streamside buffer strip) and indirect (e.g., the opportunity cost of sales lost by installing such a strip on otherwise productive cropland). Moreover, valuation includes not simply the monetary value of a provided service but also what society (consumers) might be willing to pay through mechanisms such as higher food prices or taxes.

Knowledge of the services themselves requires a fundamental understanding not only of the biophysical basis for the service but also of how different ecological processes interact to either synergize or offset the provisioning of different services: Farming is a systems enterprise with multiple moving parts and sometimes complex interactions. No-till practices, for example, can sequester soil carbon and reduce fossil fuel consumption but require more herbicide use and can increase the production of nitrous oxide (N_2_O; van Kessel et al. 2013), a potent greenhouse gas. Understanding the basis for such trade-offs and synergies requires an ecological-systems approach absent from most agricultural research.

Since 1988, we have pursued research to understand the fundamental processes that underpin the productivity and environmental performance of important row-crop systems of the upper Midwest. Our aim is to understand the key ecological interactions that constrain or enhance the performance of differently managed model cropping systems and, therefore, to provide insight into the provisioning of related services in a whole-systems context. Our global hypothesis is that ecological knowledge can substitute for most chemical inputs in intensively managed, highly productive, annual row crops. Together, long-term observations and experiments at both local and landscape scales uniquely inform our analysis.

## Experimental context: The search for services

The Main Cropping System Experiment (MCSE) of the Kellogg Biological Station (KBS), a member site of the US Long Term Ecological Research (LTER) Network, was initiated in 1988 in southwest Michigan. The site is in the US North Central Region, a 12-state region that is responsible for 80% of US corn (*Zea mays*) and soybean (*Glycine max*) production and 50% of the US wheat (*Triticum aestivum*) crop (NASS 2013a). The Great Lakes portion of the region is also an important dairy region, with alfalfa (*Medicago sativa*) being an important forage crop. Crop yields in Kalamazoo County, which surrounds the KBS LTER site, are similar to national average yields (NASS 2013a, 2013b). The soils of the area are typic hapludalfs of moderate fertility, formed since the most recent glacial retreat approximately 18,000 years ago, and the climate is humid continental (1027 millimeters [mm] per year average precipitation, 9.9 degrees Celsius mean annual temperature).

In 1988, we established a cropping-systems experiment along a management intensity gradient that, by 1992, included four annual and three perennial cropping systems plus four reference communities in different stages of ecological succession. The annual cropping systems are corn–soybean–winter wheat rotations managed in four different ways. One system is managed conventionally, on the basis of current cropping practices in the region, including tillage and, since 2009, genetically engineered soybeans and corn. One is managed as a permanent no-till system, otherwise identical to the conventional system. A third is managed as a reduced-input system, with about one-third of the conventional system's chemical inputs. In this system, winter cover crops provide additional nitrogen, and mechanical cultivation was used to control weeds until a 2009 shift to herbicide-resistant crops that allowed the use of the herbicide glyphosate for weed control in soybeans and corn. A fourth system is managed biologically, with no synthetic chemicals (or manure) but with cover crops and mechanical cultivation as in the reduced-input system. This system is US Department of Agriculture–certified organic. The three perennial crops are continuous alfalfa, short-rotation hybrid poplar trees (*Populus* var.), and conifer stands planted in 1965.

The successional reference communities include a set of early-successional sites abandoned from cultivation in 1989 and undisturbed except for annual burning to exclude trees, a set of midsuccessional sites cleared from forest in 1960 and mown annually but never tilled, a set of midsuccessional sites released from farming in the 1950s and 1960s that is now becoming forested, and a set of late-succession eastern deciduous forest stands never cleared for agriculture. Complete descriptions of each system and community appear in Robertson and Hamilton (2014).

## Delivering ecosystem services

We identify five major ecosystem services that our annual cropping systems could potentially provide: food and fuel, pest control, clean water, climate stabilization through ­greenhouse gas mitigation, and soil fertility. These services are provided to differing degrees in different systems and interact in sometimes unexpected ways. In many respects, however, their delivery comes in bundles that can be highly complementary.

### 

#### Providing food, fuel, and fiber.

Without question, the most important ecosystem service of agriculture is the provision of food; fiber; and, more recently, fuel. To an ever-increasing extent, we are dependent on high yields from simplified, intensively managed row-crop ecosystems for this provisioning. But to what extent do high yields depend on current common management practices? The results from other long-term experiments (e.g., Drinkwater et al. 1998) suggest that more-complex rotations using fewer inputs can provide similar or greater yields than those of conventional rotations. Our results suggest that simpler rotations of major grains can be managed to provide other ecosystem services, as well.

**Figure 1.**
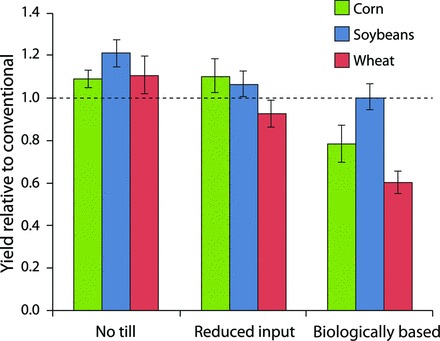
Grain yields at the Kellogg Biological Station under no-till, reduced-input, and biologically based management relative to conventional management (the dotted horizontal line) over the 23-year period of 1989–2012. The absolute yields for conventional management were similar to the county and US national average yields. The error bars represent the standard error.

Corn and soybean yields under conventional management at the KBS LTER site are similar to the average yields for both the entire United States and Kalamazoo County; wheat yields are higher (Robertson and Hamilton 2014). In our reduced-input system, corn and soybean yields slightly exceed those of our conventionally managed system, and wheat yields lag only slightly (figure 1). Indirect evidence points to nitrogen deficiency as the cause of the depressed wheat yields: Whereas corn follows a nitrogen-fixing winter cover crop and soybeans fix their own nitrogen, fall-planted wheat immediately follows the soybean harvest, which leaves relatively little nitrogen-rich residue for the wheat crop. This nitrogen deficit is especially apparent in the biologically based system, which lacks fertilizer nitrogen inputs: Wheat yields are approximately 60% of the yields under conventional management. This is in contrast to soybean yields, for which the biologically based system is equivalent to the conventional system (figure 1).

Rotational diversity clearly matters to the delivery of ecosystem services, including yield (Smith RG et al. 2008). A characteristic of intensive row-crop agriculture is its severe reduction of plant diversity of both crops and weeds. The conventional norm for most grain and other major commodity crops in the United States is weed-free monocultures or simple two-crop rotations. In the Midwest, corn is grown in a corn–soybean rotation on approximately 60% of corn acreage and in a continuous corn-only rotation on approximately 25% (Osteen et al. 2012). Simplified rotations date from the onset of highly mechanized agriculture in the 1940s. Until 1996, US farm subsidies were linked to the area planted in selected crops (notably, wheat, corn, and other feed grains), which tended to encourage simplified rotations. Today, there are two federal programs that favor simpler rotations. The most important one is the 2007 legislative mandate to blend grain-based ethanol—made entirely from corn—into the national gasoline supply. This raises demand for corn and therefore its price, creating an incentive to increase its presence in crop rotations. The second is crop insurance subsidies that reduce farmer incentives to manage risk through crop diversity.

Simplified rotations and larger fields lead to simplified landscapes, because total cropland becomes constrained to two or three dominant species in ever-larger patches (Meehan et al. 2011, Wright and Wimberly 2013). Plant diversity is further constrained by increasingly effective weed control, with chemical technologies dating from the 1950s and genomic technologies dating from the 1990s. In 2011, 94% of US soybean acreage and 70% of US corn acreage were planted with herbicide-resistant varieties (Osteen et al. 2012).

Reduced plant diversity at both the field and the landscape scales can have negative consequences for many other taxa—most notably, arthropods; vertebrates; and, possibly, microbes and other soil organisms. The loss of these taxa can have important effects on community structure and dynamics—most notably on species extinctions and changes in trophic structure that can affect pest suppression—and on ecosystem processes, such as carbon flow and nitrogen cycling. To what extent might greater rotational complexity provide these important ecosystem services?

That continuous monocultures suffer a yield penalty that persists even in the presence of modern chemicals is well known. For millennia, agriculturalists have used multispecies rotations to improve yields by advancing soil fertility and suppressing pests and pathogens (Karlen et al. 1994, Bennett et al. 2012). Since the 1950s, monoculture penalties in grain crops have been largely ameliorated with chemical fertilizers and pesticides; the remaining penalties, which appear mainly from soil pathogens or other microbial factors (Bennett et al. 2012), are largely addressable with simple two-species rotations, such as corn and soybeans.

To what extent might the restoration of rotational complexity in row crops substitute for today's use of external inputs? This is a fundamental question that underpins the success of low chemical input farming. As was noted above, the inclusion of legume cover crops plus mechanical weed control in our reduced-input corn–soybean–wheat rotation alleviated the need for two-thirds of the synthetic nitrogen and herbicide inputs otherwise required for high yields ­(figure 1). Can rotational complexity substitute for the provision of all synthetic inputs? In our biologically based system, only soybeans, which provide their own nitrogen, matched the yields of crops managed with synthetic chemicals. In organic agriculture, manure or compost is generally required to achieve high yields in nonleguminous crops (e.g., Liebman et al. 2013). However, in another experiment at the KBS LTER site, designed specifically to address the impact of rotational diversity on yield in the absence of confounding management practices, Smith RG and colleagues (2008) found that a 3-year, six-species rotation of corn, soybeans, and wheat, with three cover crops to provide nitrogen, could produce corn yields as high as the county average. In addition to yield, rotational complexity benefits other ecosystem services, as we will discuss below.

#### Providing pest protection through biocontrol services.

Biodiversity at the landscape scale also affects the capacity of agriculture to deliver ecosystem services, especially those related to biocontrol and water quality. For example, ladybird beetles (Coleoptera: Coccinellidae) are important predators of aphids in field crops. In KBS LTER soybeans, ladybird beetles are responsible for most soybean aphid (*Aphis glycines*) control and are able to keep aphid populations below economic thresholds (Costamagna and Landis 2006); absent such control, soybean yields can be suppressed 40%–60%. Coccinellid diversity is an important part of this control.

**Figure 2.**
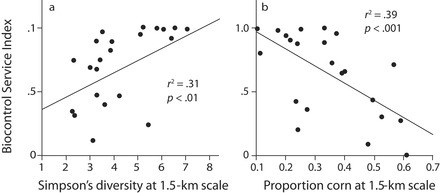
Biocontrol services from coccinellids as a function of landscape diversity (a) and the dominance of corn within 1.5 kilometers (km) of soybean fields (b). Sources: Panel (a) is adapted from Gardiner and colleagues (2009); panel (b) is reprinted from Landis and colleagues (2008).

Because different coccinellid species use different habitats at different times for foraging or other purposes, such as overwintering, the diversity of habitats within a landscape becomes a key predictor of biocontrol efficacy (figure 2a). About a dozen coccinellid species with moderate to strong habitat preferences are present in the KBS landscape (Maredia et al. 1992a, Landis and Gage 2014). *Coleomegilla septempunctata*, for example, overwinters in woodlots and, prior to the summertime development of soybean aphid populations, depends on pollen from early flowering plants, such as Virginia springbeauty (*Claytonia virginica* L.) and the common dandelion (*Taraxacum officinale* F.H. Wigg.), and then on aphids in the winter wheat and alfalfa crops (Colunga-Garcia 1996). Later in the season, after aphids have fed on soybeans, the early-successional and poplar communities support late-season aphid infestations that are exploited by the coccinellids (Maredia et al. 1992b).

Landscape diversity can therefore be key for biocontrol services provided by mobile predators. For coccinellids, the presence of heterogeneous habitats within 1.5 kilometers of a soybean field is strongly correlated with soybean aphid suppression: Landscapes with greater proportions of the local area in corn and soybean production have significantly less biocontrol (figure 2b; Gardiner et al. 2009). Landis and colleagues (2008) estimated the value of hidden biocontrol in Michigan and three adjacent states to be $239 million for 2007 on the basis of a $33 per hectare (ha) increase in profitability from higher production and lower pesticide costs among the soybean farmers who used integrated pest management to control aphids.

#### Providing clean water.

The quality of water draining from agricultural watersheds is a longstanding environmental problem. Sediment, phosphorus, and nitrate are important pollutants that leave cropland and lead to compromised groundwater, surface freshwaters, and marine ecosystems worldwide. In the United States, over 70% of the nitrogen and phosphorus delivered to the Gulf of Mexico by the Mississippi River is derived from agriculture (Alexander et al. 2008). Such deliveries create coastal hypoxic zones worldwide (Diaz and Rosenberg 2008).

Must this necessarily be the case? Sediment and phosphorus loadings can be reduced substantially with appropriate management practices: No-till and other conservation tillage methods can often eliminate erosion and substantially reduce the runoff that also carries phosphorus to surface waters, as can riparian plantings along cropland waterways (Lowrance 1998). Nitrate mitigation is more problematic. Because nitrate is so mobile in soil, percolating water carries it to groundwater reservoirs, where it resides for days to decades before it emerges in surface waters and is then carried downstream (Hamilton 2012), eventually to coastal marine systems.

Some of the transported nitrate can be captured by riparian communities (Lowrance 1998) or can be processed streamside (Hedin et al. 1998) or in transit (Beaulieu et al. 2011) to more reduced forms of nitrogen, including nitrogen gas. If wetlands are in the flow path, a significant fraction can be immobilized in wetland sediments as organic ­nitrogen or can be denitrified into nitrogen gas, either by hetero­trophic or chemolithoautotrophic microbes (Whitmire and Hamilton 2005, Burgin and Hamilton 2007). Restoring wetlands and the tortuosity of more-natural channels can increase both streamside and within-stream processing of nitrate (NRC 1995).

But, by far, the best approach to mitigating nitrate loss is avoiding it to begin with—a major challenge in cropped ecosystems so dependent on large quantities of plant-available nitrogen. The average nitrogen fertilizer rate for corn in the Midwest is approximately 160 kilograms (kg) of nitrogen per ha (ERS 2013), with only about 50% taken up by the crop, on average (Robertson 1997). This contrasts with annual inputs of approximately 7 kg of nitrogen per ha delivered in precipitation at the KBS LTER site.

**Figure 3.**
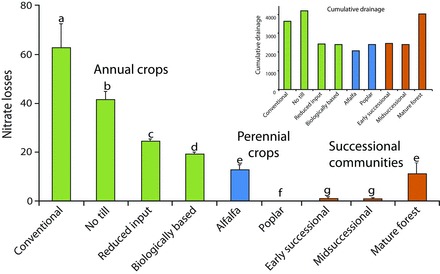
Annual nitrate leaching losses (in kilograms of nitrate-nitrogen per hectare per year) and cumulative drainage (inset; in millimeters) from the Kellogg Biological Station cropping and successional systems between 1995 and 2006. Source: Adapted from Syswerda and colleagues (2012).

KBS LTER research has shown that crop management can substantially reduce long-term nitrate leaching. Over an 11-year period, beginning 6 years after establishment, the MCSE annual row-crop systems showed two- to threefold differences in nitrate losses, ranging from average annual losses of 19 and 24 kg of nitrogen per ha in the biologically based and reduced-input systems, respectively, and of 42 and 62 kg of nitrogen per ha in the no-till and conventionally managed systems, respectively (figure 3; Syswerda et al. 2012). Even after accounting for yield differences (figure 1), leaching differences were substantial: 7.3 kg of nitrate-nitrogen per megagram (Mg) yield in the reduced-input system, compared with 11.1 in the no-till and 17.9 in the conventional systems.

What accounts for lower nitrate-leaching rates? The better soil structure in no-till cropping systems allows water to leave more quickly (Strudley et al. 2008), which reduces equilibration with soil microsites where nitrate is formed. But a more important factor appears to be the presence of cover crops: Even with tillage, the reduced-input and biologically based systems leached less nitrogen. Cover crops helped ­perennialize the crop year; that is, with the fields occupied by growing plants for a greater proportion of the year, more nitrate is scavenged from the soil profile and cycled through plant and microbial transformations (McSwiney et al. 2010). More soil water is also transpired, which reduces the opportunity for nitrate transport: Drainage in the reduced-input and biologically based systems was only 50%–70% of that in the conventional and no-till systems (figure 3 inset). The rapidly growing systems with true perennial vegetation—the poplar and successional systems—had exceedingly small annual leaching rates of 0.1–1.1 kg of nitrogen per ha, although that was, in part, due to very low or nonexistent rates of nitrogen fertilizer use. In a related experiment, a perennial cereal crop fertilized at agronomic levels leached 80% less nitrate than did its annual analogue (Culman et al. 2013).

#### Providing greenhouse gas mitigation.

Agriculture is directly responsible for approximately 10%–14% of total annual global anthropogenic greenhouse gas emissions (Smith P et al. 2007). This is largely the result of N_2_O emitted from soil and manure and from methane emitted by ruminant animals and burned crop residues. Including the greenhouse gas costs of agricultural expansion, agronomic inputs, such as fertilizers and pesticides, and postharvest activities, such as food processing, transport, and refrigeration, bring ­agriculture's footprint to 26%–36% of all anthropogenic greenhouse gas emissions (Barker et al. 2007). Mitigating some portion of this footprint could therefore significantly contribute to climate stabilization (Caldeira et al. 2004), as might the production of cellulosic biofuels if they were used to offset fossil fuel use (Robertson et al. 2008).

**Figure 4.**
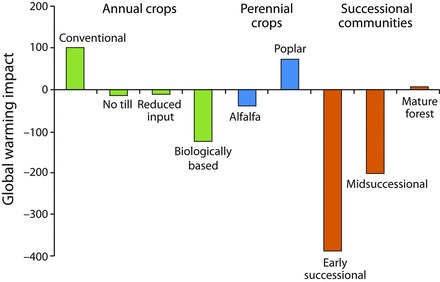
Net global warming impact (in grams of carbon dioxide equivalent per square meter per year) of cropped and unmanaged Kellogg Biological Station ecosystems. The annual crops include corn–soybean–wheat rotations.

Global warming impact analyses can reveal the source of all significant greenhouse gas costs in any given ­cropping system and, therefore, the full potential for management to mitigate emissions. Such an analysis for KBS LTER cropping systems over a 20-year time frame (figure 4; Gelfand and Robertson 2014) shows how the overall costs can vary substantially with management. The conventional annual cropping system had a net annual global warming impact (in carbon dioxide equivalents [CO_2_e]) of 101 grams (g) of CO_2_e per square meter (m^2^), whereas the no-till system exhibited net mitigation: –14 g of CO_2_e per m^2^. The early-successional system was the most mitigating, at –387 g of CO_2_e per m^2^. Closer inspection reveals the basis for these differences: Although N_2_O production and nitrogen fertilizer manufacture were the two greatest sources of global warming impact in the annual cropping systems, the soil carbon storage in the no-till system more than offset the CO_2_e cost of no-till N_2_O and fertilizer manufacture. And because the biologically based system sequestered carbon at an even greater rate and without the added cost of nitrogen fertilizer, the net mitigation was stronger still (figure 4; Gelfand and Robertson 2014).

**Figure 5.**
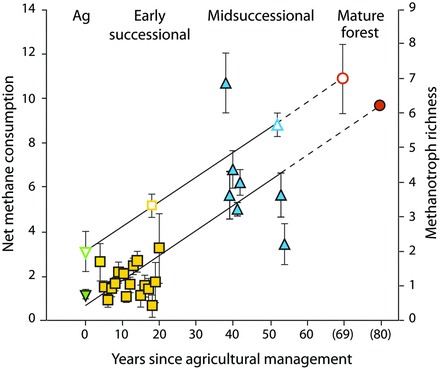
The increase in soil methanotroph diversity (in operational taxonomic units; the open symbols) and atmospheric methane consumption (in grams of methane-carbon per hectare per day; the closed symbols) in ecological succession from row-crop fields (Ag, green) through early (yellow) and midsuccessional (blue) fields to mature forest (orange) at the Kellogg Biological Station. Source: The data are from Levine and colleagues (2011).

Most of the substantial mitigation capacity of early-successional fields is derived from their high rate of soil carbon storage, which will diminish over time. At the KBS LTER site, the carbon stored annually in midsuccessional soils was approximately 10% of that in early-succession soils, and no net soil carbon storage occurred in the mature deciduous forest. As a result, the net CO_2_e balance of the mature forest is close to 0 g of CO_2_e per m^2^, with methane oxidation offsetting most of the CO_2_e cost of natural N_2_O emissions (figure 4). Interesting, too, is the recovery of methane oxidation during succession. Methane oxidation rates are typically decimated when natural vegetation is converted to agriculture (Del Grosso et al. 2000); that oxidation in the midsuccessional system is more than midway between that of the early-successional system and that of the mature forest suggests an 80–100-year recovery phase (figure 5). Recent evidence from the KBS LTER site suggests that methanotrophic bacterial diversity plays a role in methane oxidation differences (figure 5; Levine et al. 2011).

In addition, if harvested biomass is used to produce energy that would otherwise be provided by fossil fuels, the net global warming impact of a system will be further reduced by avoided carbon dioxide emissions from the fossil fuels displaced by the biomass-derived energy. Sometimes—as with corn grain in conventional systems—the displacement is minor or even nonexistent because of the fossil fuel used to produce the biomass (Farrell et al. 2006) and the potential to incur carbon costs elsewhere by clearing land to replace that removed from food production (Searchinger et al. 2008). In contrast to the energy provided by corn grain is the energy provided by cellulosic biomass produced in the early-successional system. Gelfand and ­colleagues (2013) calculated that harvesting successional vegetation for cellulosic biofuel could provide approximately 850 g of CO_2_e per m^2^ of greenhouse gas mitigation annually. Extrapolated yields to marginal lands across 10 Midwest states using finescale (0.4-ha) modeling yielded a potential climate benefit of approximately 44 million metric tons of carbon dioxide per year. However, such near-term benefits also depend on the methods used to establish the biofuel crop; killing the existing vegetation and replanting with purpose-grown feedstocks, such as switchgrass or miscanthus, can create substantial carbon debt (Fargione et al. 2008) that can take decades to repay (Gelfand et al. 2011); the debt is even greater if the replanted crop requires ­tillage (Ruan and Robertson 2013).

The provision of greenhouse gas mitigation is a service clearly within the capacity of modern cropping systems to provide. Various management practices have differing effects, sometimes in opposition (consider, e.g., no-till energy savings versus the carbon cost of additional herbicides) and at other times synergistic (consider that leguminous cover crops in the biologically based systems not only increased soil carbon storage but also reduced the CO_2_e costs of manufactured fertilizer nitrogen). Designing optimal systems is not difficult; there are many practice-based opportunities to diminish CO_2_e sources or enhance CO_2_e sinks and thereby help stabilize the climate.

#### Providing soil fertility, the basis for sustained crop production.

Closely tied to other services, such as food production and greenhouse gas mitigation, is soil fertility. As a supporting service that underpins the provision of other services (MA 2005), soil fertility is under management control and is therefore a deliverable service; in its absence, fertility must be enhanced with greater quantities of external inputs, such as fertilizers, and the system is less able to withstand extreme events, such as drought. That said, soil fertility is not a panacea for reducing the environmental impacts of agricultural systems; for example, N_2_O production was as high in our biologically based system as it was in the less fertile conventional system (Robertson et al. 2000).

Soil fertility has many components. Physically, fertility is related to soil structure—porosity, aggregate stability, water-holding capacity, and erosivity. Its chemical constituents include soil organic matter, pH, base saturation, cation exchange, and nutrient pools. Biologically, soil fertility is related to food web complexity, pest and pathogen suppression, and the delivery of mineralizable nutrients. Most of these components are interrelated, which frustrates attempts at a comprehensive definition of *soil fertility* or *soil quality*. At heart, however, *soil fertility* is the capacity of a soil to meet plant growth needs; all else equal, more-fertile soils support higher rates of primary production.

Building soil fertility is closely tied to building soil organic matter: A century of work at Rothamsted and other long-term agricultural research sites (Rasmussen et al. 1998) has shown positive associations with most—if not all—of the indicators noted above. At the KBS LTER site, relative to the conventional system, soil organic matter increased in the ­no-till, reduced-input, and biologically based systems (Syswerda et al. 2011). A major reason for soil carbon gain in these systems is slower decomposition rates as a result of organic matter protection within soil aggregates, particularly within larger size classes. Grandy and Robertson (2007) found greater soil carbon accumulation in KBS LTER ecosystems with higher rates of large (2–8 millimeters) aggregate formation. The formation of large aggregates and carbon accumulation was ­greatest in the successional and mature forest systems and lowest in the conventional system; the biologically based, no-till, and perennial systems were intermediate. Aggregates in smaller size classes (up to 0.25 ­millimeters) expressed the opposite trend.

That the no-till system accumulated carbon and primarily in larger, more vulnerable aggregates is no surprise (West and Post 2002, Six et al. 2004); however, carbon and large aggregate accumulation in the heavily tilled reduced-input and biologically based systems was unexpected and likely related to the inclusion of leguminous cover crops in these rotations. Legumes may increase aggregate stability through greater polysaccharide production and different microbial communities (Haynes and Beare 1997).

**Figure 6.**
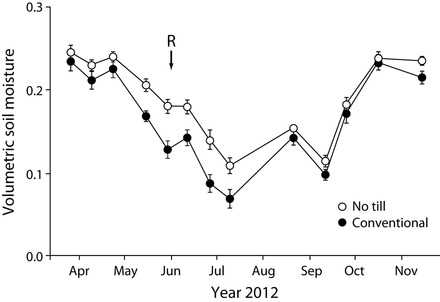
Seasonal variation in soil moisture (in cubic centimeters per cubic centimeter) in the conventional and no-till systems during the 2012 soybean growing season. The 6-week drought began after a 3 June rainfall (R on the figure). The error bars represent the standard error (*n* = 6).

That the no-till system better withstood the 2012 US drought than did the other systems (no-till system, mean = 1.9 Mg per ha of soybean grain, standard error of the mean [SEM] = 0.12; conventional system, mean = 1.3 Mg of soybean grain per ha, SEM = 0.05) suggests a clear no-till benefit to soil fertility even when external inputs are high. Greater moisture stores in the better-structured no-till soils following the last significant rainfall before the drought (figure 6), equivalent to approximately 4 centimeters of stored water in the root zone, underscores the value of no-till agriculture to the 2012 soybean production. This enhanced water storage capacity may also help explain greater no-till productivity in more normal years; on average, yields in the no-till system were 9%–21% higher than they were in the conventional system (figure 1). In the reduced-input system, soil fertility allowed competitive yields (figure 1) with only a fraction of the nitrogen and other inputs.

## Valuing ecosystem services: The social component

The ability of row crops and agricultural landscapes to provide ecosystem services is only part of the farming for services equation. The other is farmers’ willingness to implement practices that deliver additional services and, to the extent that adoption probably requires economic compensation, society's willingness to pay for these services.

The willingness of farmers to adopt new management practices that provide additional services depends on awareness, attitudes, available resources, and incentives (Swinton et al. 2014a). The current practices are largely the result of past practices; cultural norms; and the availability of technology, policies, and markets that support sustained profitability. Although environmental stewardship is a factor influencing many farmers’ decisions, sustained profitability is usually the overriding concern.

**Figure 7.**
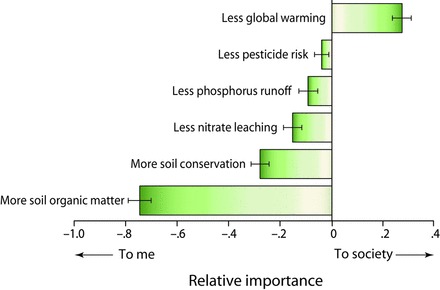
The relative importance to Michigan farmers and to society (as ranked by the farmers) of various environmental benefits potentially provided by agriculture. Source: Adapted from Swinton and colleagues (2014a).

Particularly for those services related to reducing the environmental impact of agriculture, farmers in Michigan—and presumably elsewhere—are more likely to adopt practices that provide direct, local benefits. These benefits might be monetary, such as higher profits or greater future land values, or nonmonetary, such as safer groundwater for family use. To learn how farmers weigh environmental benefits in their management ­decisions, Swinton and colleagues (2014a) conducted a series of six farmer focus groups in 2007 and a subsequent statewide survey of 1600 Michigan corn and soybean farms in 2008 (Jolejole 2009, Ma et al. 2012). When asked to consider six environmental benefits of reduced-input agriculture and to rate their relative importance to themselves and to society, the participating farmers in both settings ranked benefits such as increased soil organic matter, soil conservation, and reduced nitrate leaching as significantly more important to themselves than to society (figure 7). In contrast, reduced global warming was ranked as more important to society than to the farmers. These attitudes conform to the economic distinction between private and public goods and will strongly influence the farmers’ willingness to accept payments for shouldering a perceived public burden.

The survey revealed that, of a variety of cropping practices known from KBS and other research to provide environmental benefits, two practices were currently used by over 80% of the participants (Swinton et al. 2014a). These included pest scouting prior to insecticide application and reduced tillage (e.g., chisel plowing). These practices saved labor or inputs or improved farmstead water quality without reducing expected crop revenue; they were therefore desirable with respect to both the environment and farm profitability.

A second group of three practices was viewed favorably by about half of the farmers: the addition of a small grain, such as wheat, to their standard corn–soybean rotation; incorporating rather than spreading manure; and no-till management, at least for specific crop years. In comparison to the first group of practices, these were perceived to have a greater risk of diminished revenues, higher costs, or greater labor demands during busy periods.

A third group of practices appealed to less than a third of the farmers: continuous no-till, banded application of fertilizer and pesticides at reduced rates, soil testing for nitrogen prior to nitrogen fertilization, and winter cover crops to substitute for most fertilizer nitrogen; these practices were seen as of particularly high risk. This result reveals that, for the rotation tested, although higher average yields (figure 1) and comparable profitability (Jolejole 2009) may be apparent under experimental conditions, they are not by themselves sufficient for the adoption of reduced-input management.

A separate section of the survey elicited what levels of payment (if any) the farmer respondents would require to adopt environmentally beneficial cropping practices. Farmers were presented with four increasingly demanding cropping systems with similarly increasing environmental benefits that ranged from a chisel-tilled corn–soybean rotation fertilized according to university recommendations (including a nitrate soil test) to a corn–soybean–wheat rotation with winter cover crops and reduced chemical inputs. The respondents were asked how much land they might enroll in each system for a predetermined payment level.

Three farmer traits—the belief that their production could benefit from nature, their years of prior experience, and the availability of suitable equipment—were collectively the best predictor of farmers’ willingness to shift land into the more complex cropping systems associated with reduced chemical inputs (Ma et al. 2012). Not surprisingly, the simplest system attracted the most participation, regardless of farm size. However, among those willing to adopt the most environmentally beneficial system, farmers with over 200 ha were much more willing than were farmers with smaller farms to offer more acreage at higher payment levels. These larger farms are therefore most likely to be providers of environmental services at the lowest cost (Swinton et al. 2014b). This is probably related to economies of scale: Not only do larger farms have more land to enroll, but the additional fixed costs of no-till, banding, and cultivation equipment can be spread out over larger areas, therefore lowering capitalization barriers.

Clear from this research is that the provision of ecosystem services in agriculture will require incentives. Education is not the issue; most farmers are aware of the environmental benefits of alternative practices (except for greenhouse gas mitigation benefits). Indeed, those farmers who strongly valued environmental stewardship were willing to accept lower cost incentives to adopt the alternative practices (Ma et al. 2012). But almost all of the farmers—especially those with large farms—were willing to accept payments for services. This, then, raises the question *Are consumers ­willing to pay for such services?* Regardless of the mechanisms whereby payments are made—direct payments to farmers through government or private programs, tax abatements, or higher prices to consumers from taxes on polluting inputs or ­tradable pollution credits (Lipper et al. 2009)—the cost of payments for ecosystem services must ultimately be borne by society.

The 2009 Michigan Environmental Survey (Chen 2010, Swinton et al. 2014b) provides insight on society's willingness to pay. The survey was returned by approximately 2400 households from every county in Michigan, stratified by population. The respondents were asked about their willingness to support a personal income tax increase to pay land managers to enroll in one of three stewardship programs that would, to varying degrees, reduce lake eutrophication or atmospheric greenhouse gas concentrations.

The responses to the survey showed substantial public willingness to finance policies that would pay farmers to adopt practices to abate lake eutrophication. In aggregate, the respondents were willing to pay $175 per household for a combined reduction of 170 eutrophic lakes and 0.5% lower greenhouse gas emissions. However, most of the households were unwilling to pay farmers for reduced greenhouse gas emissions alone. Over 60% of the households in 2009 were unconcerned about climate change. Of the 40% that were concerned, however, the mean household was willing to pay $141 per year for a 1% reduction per year in greenhouse gas emission levels.

On the supply side, then, the Michigan corn and soybean farmers were clearly willing to change their cropping practices to generate additional ecosystem services if they were paid to do so. The farmers would expand both the complexity of their farming practices and the acreage under these practices if they were given the opportunity and would thereby generate a supply of land managed to deliver additional ecosystem services. On the demand side, the state residents appeared to be willing to pay for reduced numbers of eutrophic lakes, and a significant fraction of the residents appeared to be willing to pay for reduced greenhouse gas emissions. How can we link buyers and sellers?

Important to both groups was how ecosystem services are characterized and bundled. It is difficult to measure the value of individual ecosystem services from agriculture. Management decisions affect multiple services simultaneously; farming is a systems-level enterprise with system-level responses (Robertson et al. 2004), such that ecosystem services come in bundles and should probably be marketed as such. Credit stacking in carbon and other payment for environmental services markets (e.g., Fox 2011) cannot be avoided because of the varied objectives of the many willing governmental and nongovernmental payers. Moreover, credit stacking should probably be encouraged in order to take full advantage of cobenefits and fully exploit available synergies. Converting demand for additional ecosystem services into the area of land required to generate the desired change is a logical next step.

Although approaches to payment for ecosystem services deserve further research, they are but one among many policy tools available to meet the demand for additional ecosystem services. Exploring and testing alternative tools—especially in light of new precision-farming technologies—is an appropriate response to the evidence here that, at reasonable prices, farmers are willing to supply and consumers are willing to pay for a meaningful set of ecosystem services.

## Where from here?

Additional knowledge about row-crop ecosystems will reveal additional opportunities for providing services and delivering them more efficiently. One example might be to manage noncrop areas in agricultural landscapes to support natural enemies of crop pests (Landis et al. 2000). Another might be to more precisely estimate or meet crop nitrogen needs in order to avoid excess nitrogen fertilizer additions (Robertson and Vitousek 2009). And a third might be to manage the soil microbial community to restore the capacity to remove methane from the atmosphere (Levine et al. 2011). Understanding and evaluating the delivery of services in a systems context will allow the full suite of trade-offs and synergies to be considered.

Long-term agricultural research reveals ecological trends that build slowly and sometimes subtly. It also allows researchers to capture the expression of episodic events, such as weather extremes, pest outbreaks, and species introductions, and it permits the evaluation of biological change against slow but steady changes in climate; technology; markets; and public attitudes toward food, fuel, and the environment. In the coming decades, human population and income growth will drive agriculture to ever-higher intensities. Now is the time to guide this intensification in a way that enhances the delivery of ecosystem services that are not currently marketed. Delaying action will result in an environment further degraded and an agriculture that is further divorced from its biological roots, more vulnerable to climate extremes and pest outbreaks, and increasingly dependent on external energy and synthetic chemical inputs.

Systems-level research reveals how disparate parts of agricultural ecosystems interact in subtle, often surprising, and sometimes crucial ways. Connections among microbial community structure, the formation of soil organic matter, soil water-holding capacity, plant drought tolerance, and primary productivity and herbivory are difficult to detect in the absence of research in which multiple parts of the same system are studied simultaneously. And research that is too local and that fails to consider relationships among different cropped and noncropped habitats within the larger landscape will likewise fail to make apparent crucial opportunities for designing future cropping systems that are productive, resilient, and able to deliver a rich suite of ecosystem services. Systems-level research sufficiently reductionist to identify key organism-level interactions and processes will be increasingly valuable for delivering worthwhile opportunities.

Some of these opportunities will be more generalizable than others. They will all require adaptations to local environmental and economic conditions, and both policy and research must include the need for flexible solutions, especially as new genomic and other technologies enter the marketplace. Trade-offs and synergies must be recognized and evaluated (e.g., Syswerda and Robertson 2014) in order to design optimal systems for specific outcomes. Ultimately, modeling will be needed to help design specific solutions for specific locales.

Research from the KBS LTER site reveals a number of worthwhile opportunities for delivering services today. Almost all of those opportunities are interdependent. Some of these interdependencies are synergistic, suggesting multiple paths for farmer adoption; others are negative, suggesting the need for targeted incentives for particular services important to society. Identifying such interdependencies and how they respond to different management practices and environmental change is a need in cropping systems everywhere and has never been more urgent.
